# Mild phenotype of glutaric aciduria type 1 in polish patients – novel data from a group of 13 cases

**DOI:** 10.1007/s11011-018-0357-5

**Published:** 2018-12-20

**Authors:** Paulina Pokora, Aleksandra Jezela-Stanek, Agnieszka Różdżyńska-Świątkowska, Elżbieta Jurkiewicz, Anna Bogdańska, Edyta Szymańska, Dariusz Rokicki, Elżbieta Ciara, Małgorzata Rydzanicz, Piotr Stawiński, Rafał Płoski, Anna Tylki-Szymańska

**Affiliations:** 1 Department of Pediatrics, Nutrition and Metabolic Diseases, The Children’s Memorial Health Institute, Warsaw, Poland; 2 Department of Genetics and Clinical Immunology, National Institute of Tuberculosis and Lung Diseases, Warsaw, Poland; 3 State Tertiary Vocational School in Oswiecim, Oswiecim, Poland; 4 Anthropology Laboratory, The Children’s Memorial Health Institute, Warsaw, Poland; 5 Department of Diagnostic Imaging, The Children’s Memorial Health Institute, Warsaw, Poland; 6 Department of Biochemistry, Radioimmunology and Experimental Medicine, The Children’s Memorial Health Institute, Warsaw, Poland; 7 Department of Medical Genetics, The Children’s Memorial Health Institute, Warsaw, Poland; 8 Department of Medical Genetics, Medical University of Warsaw, Warsaw, Poland

**Keywords:** Glutaric aciduria type 1, *GCDH* gene, Phenotype-genotype correlation

## Abstract

Glutaric aciduria type 1 is a neurometabolic disorder, caused by riboflavin-dependent glutaryl-CoA dehydrogenase deficiency. As its consequence, accumulation of the putatively neurotoxic metabolites (glutaric and 3-hydroxyglutaric acids) in body tissues, but especially within the brain, is observed. Estimated incidence of the disease is 1 in 110,000 newborns, The prevalence however may be higher, depending on a specific ethnic group, and result in phenotypic variation as well. In this paper we present clinical data of 13 patients of Polish nationality. They all present a mild phenotype and clinical course of glutaric aciduria type 1. Based on their clinical data, presented herein, we like to pay attention to the phenotypic and neuroimaging features important for the diagnosis of mild form of this disease. Moreover, we present novel molecular data, which may correlate with such a manifestation.

**Electronic supplementary material:**

The online version of this article (10.1007/s11011-018-0357-5) contains supplementary material, which is available to authorized users.

## Introduction

Glutaric aciduria type 1 (GA1) is caused by riboflavin-dependent glutaryl-CoA dehydrogenase (GCDH) deficiency, encoded by the *GCDH* gene (localized on chromosome 19p13.2). The enzyme converts glutaryl-CoA to crotonyl-CoA in the lysine, hydroxylysine, and tryptophan metabolism (Nyhan and Ozand 1998). As a result of its deficiency accumulation of the putatively neurotoxic metabolites (glutaric and 3-hydroxyglutaric acid, GA and 3-OH-GA) in body tissues, especially within the brain is observed. Estimated incidence of GA1 is 1 in 110,000 newborns (Boy et al. 2017). The prevalence however may be higher in the specific ethnic groups (Haworth et al. 1991). Similarly, the clinical course can vary depending on the ethnic group.

Based on a clinical outcome three clinical subtypes of GA1 were proposed: acute, (majority patients), insidious (about one third of symptomatic patients) and late-onset types (asymptomatic cases of maternal GA1) (Hoffmann et al. 1996). In its acute form the disease manifests with an episode of metabolic decompensation with ketoacidosis, hyperammonemia, hypoglycemia, which almost always occur during an infectious illness, as well as encephalopathy that develops usually during the first year of life. In both acute and insidious form the affected children also develop an irreversible dystonic movement disorder (Nyhan and Ozand 1998; Hartley et al. 2001; Hoffmann et al. 1991; Gordon 2006).

One of most characteristic and widely known physical features of this aciduria is macrocephaly, observed in about 75% patients (Boy et al. 2017). It may be the earliest sign of GA1 that is seen at birth or it occurs as rapid increase of the head circumference during infancy (Nyhan, 1998; Gordon 2006; Strauss et al. 2003). In this paper we aimed to focus on glutaric aciduria type 1 phenotype, especially head MRI and circumference with relation to biochemical and genetic results (urinary excretion of glutaric and 3-hydroxyglutaric acids and causative pathogenic variants in *GCDH* gene, respectively) based on clinical data of 13 Polish patients.

## Patients and methods

### Material

Data of 13 Polish GA1 patients, from 11 families (9 females of whom there are 2 pairs of sisters, and 4 males) were analysed with a focus on age of diagnosis, first symptoms and(or) method of initial testing (target vs. newborn screening), gas chromatography mass spectrometry profile (glutaric and 3-hydroxyglutaric acids), brain imaging results (brain MRI), and *GCDH* gene mutations. The cognitive function was established according to patients’ age (as Psychomotor Development Index (PDI) in those less than 6 years old and Intellectual Quotient (IQ) for older ones).

In 3 children the diagnosis was based on newborn screening (NBS), while in the remaining 10 patients is a result of selective screening (by GC-MS). Positive family history was the trigger for testing in 2 cases. Clinical data are summarised in Table 1.

**Table 1:** Clinical and molecular data of 13 Polish patients with Glutaric aciduria type I

Patient/Gender	Age at diagnosis	Method of screening/Result	First symptoms	Clinical status and history	*GCDH* gene mutations	Genotype acc. to HGVS Protein level
1/m	4d	newborn		during infancy: slightly delayed motor development; 7 m: trembling during anxiety and when waking up; in EEG: localized changes (in observation); 2y2m: average level of development (PDI-89); can speak only single words 5y: normal development (kindergarten)	c.680G > C (p.Arg227Pro) /c.1204C > T (p.Arg402Trp)	c.[680G > C](;)[1204C > T] p.[Arg227Pro](;)[Arg402Trp]
2/f (sister of 12)	3w	newborn		17 m: unsteady gait; 5y: lymphoblastic lymphoma (successfully cured) 5y4m: speech disability (indistinct); PDI = 85, non-harmonious development, poor graphomotor skills; 7y: IQ – 104, development in the normal range; clumsy movement and slight balance disorders	c.700C > T (p.Arg234Trp) /c.700C > T (p.Arg234Trp)	c.[700C > T];[(700C > T)] p.[Arg234Trp];[(Arg234Trp)]
3/f (sister of 13)	3w	newborn/high-excretor		11 m: average level of psychomotor functioning; 4y8m: PDI = 112	c.1204C > T (p.Arg402Trp) /c.1204C > T (p.Arg402Trp)	c.[1204C > T];[(1204C > T)] p.[Arg402Trp];[(Arg402Trp)]
4/m	1 m	selectiveGC-MS/high-excretor	rapid increase of head circumference	slightly delayed speech development, howling, impaired auditory memory and learning difficulties, poor coordination; 6y: intellectual development in the normal range	c.1204C > T (p.Arg402Trp) /c.1204C > T (p.Arg402Trp)	c.[1204C > T];[(1204C > T)] p.[Arg402Trp];[(Arg402Trp)]
5/m	14y	selective GC-MS	delayed motor development, rapid increase of head circumference, coordination problems	special-needs school, then vocational (profession of carpenter), mild intellectual disability, poor language ability, headaches, discrete features of dystonia	c.937C > T (p.Arg313Trp) /c.937C > T (p.Arg313Trp)	c.[937C > T];[(937C > T)] p.[Arg313Trp];[(Arg313Trp)]
6/f	14y	selective GC-MS	enlarging fontanelle in infancy, periventricular cysts in US scan, the sunset eye sign, horizontal nystagmus. MRI (3w of life): bilateral arachnoid cyst (developmental variant), epilepsy from 1y of age	mild intelectual disability, special-needs school, balance disorders, movement clumsy, in good contact but rebellious, infantile	c.775 T > C (p.Ser259Pro) /c.1204C > T (p.Arg402Trp)	c.[775 T > C](;)[1204C > T] p.[Ser259Pro](;)[Arg402Trp]
7/f	4.5y	selective GC-MS/high-excretor	delayed motor development, excessive fatigue during exercise, balance disorders	unsteady gait with balance disorder, speech deficits, average intellect but learning difficulties (and individual schooling), hyperactivity, epilepsy	c.1204C > T (p.Arg402Trp) /c.1204C > T (p.Arg402Trp)	c.[1204C > T];[(1204C > T)] p.[Arg402Trp];[(Arg402Trp)]
8/f	15y	selectiveGC-MS/high-excretor	large head circumference in infancy	lack of neurological symptoms and sense of disease, periodic headaches, has a degree in mathematics	c.1063C > T (p.Arg355Cys) /c.1063C > T (p.Arg355Cys)	c.[1063C > T];[(1063C > T)] p.[Arg355Cys];[(Arg355Cys)]
9/f	21 m	selective GC-MS/high-excretor	at the age of 15 m: 2-times loss of consciousness, in CT: bilateral symmetrical widening of fluid cerebral spaces at the frontal poles of the temporal lobes	20 m: PDI = 106; seizures at 22 m and 3y	c.1063C > T (p.Arg355Cys) / c.1204C > T (p.Arg402Trp)	c.[1063C > T](;)[1204C > T] p.[Arg355Cys](;)[Arg402Trp]
10/f	9 m	selective GC-MS	macrocephaly; 18 m: metabolic decompression (1 epizode);	normal development (no balance disorders or seizures)	c.1064G > A (p.Arg355His) /? no mutation on second allele (only selected *GCHD* exons were analysed)	c.[1064G > A];[?] p.[Arg355His];[?]
11/m	28 m	selective GC-MS/high-excretor	delayed motor development, rapid increase of the head circumference	delayed motor and speech development, mobility clumsiness, broad-base unstedy gait, poor fine motor skils	c.1204C > T (p.Arg402Trp) /c.1204C > T (p.Arg402Trp)	c.[1204C > T];[(1204C > T)] p.[Arg402Trp];[(Arg402Trp)]
12/f (sister of 2, born from twin pregnancy – twin-brother is healthy)	1 m	selective (positive family history)/ GC-MS/high-excretor		unsteady gait; slightly clumsy movement and slight balance disorders; normal development	c.700C > T (p.Arg234Trp) / c.700C > T (p.Arg234Trp)	c.[700C > T];[(700C > T)] p.[Arg234Trp];[(Arg234Trp)]
13/f (sister of 3)	9y	selective (positive family history) GC-MS	from 7y of age: vertigo and vomiting	12y: intellectual development in the normal range; in the CBCL self-assessment questionnaire: slightly increased withdrawal rate, anxiety at the borderline level and somatic symptoms	c.1204C > T (p.Arg402Trp) /c.1204C > T (p.Arg402Trp)	c.[1204C > T];[(1204C > T)] p.[Arg402Trp];[(Arg402Trp)]

HGVS - Human Genome Variation Society; IQ - Intellectual Quotient; PDI - Psychomotor Development Index; CBCL - Child Behavior Checklist

In all diagnosed patients, in line with the current recommendation, specialized dietary treatment, carnitine and riboflavin has been applied consisting of a low-protein diet containing the minimum natural protein required for growth (Boy et al. 2017).

All patients underwent further molecular diagnostics, which verified the presence of glutaric aciduria type 1 and allowed to identify causative mutation in *GCDH* gene. Pathogenic molecular variants were identified in all patients (on 25 alleles because in one patient only selected exons were analysed and hence the mutation was found only in 1 allele, Table 1).

### Gas chromatography mass spectrometry (GC-MS) technique

Urinary concentrations of glutaric acid and 3-hydroxyglutaric were measured by GC-MS (gas chromatography - mass spectrometry) according to procedures described previously (Chalmers and Lawson 1975; Goodman and Markey 1981; Chalmers and Lawson 1982).

### Next-generation sequencing (NGS) procedure

Molecular analysis was performed after obtaining informed consent from the patients’ parents or legal guardians. The next-generation sequencing aimed to identify molecular defects in *GCDH* gene. The analysis was performed at the Department of Medical Genetics, Medical University of Warsaw according to established laboratory protocols (Ploski et al. 2014). The NGS data were analysed using an in-house procedure (Ciara et al. 2018).

The nomenclature of molecular variants follows the Human Genome Variation Society guidelines (HGVS, www.hgvs.org/mutnomen) using human *GCDH* (*608801; glutaryl-CoA dehydrogenase) cDNA sequence: NM_000159.3 followed the Human Gene Mutation Database (HGMD, www.hgmd.cf.ac.uk).

The protocol was approved by the Human-subjects Institutional Review Board at the Bioethics Committee of the Children’s Memorial Health Institute, Warsaw, Poland. A written informed consent was provided by parents or legal guardians.

## Results

Clinical data analysed in this study are presented in Table 1. The patients were classified into 3 groups based on the indication for testing. Three children were diagnosed in a newborn screening (NBS) – Group 1, two another based on positive family history (one in newborn period, second one at 9 years of age) – Group 3. Selective screening (GC-MS), performed due to observed developmental problems, allowed for diagnosing in the remaining 8 – Group 2. The mean age of the latter was 6,5 years.

### Outcome

Among 3 patients diagnosed in NBS (Group 1), development at the age about 5 years was assessed as normal in all. However, speech delay in one and poor graphomotor skills with speech disability in another were observed earlier.

In most of Polish patients diagnosed based on selective GC-MS screening (Group 2) glutaric aciduria type 1 physically manifested with rapid increase of head circumference (3/13; 23%) and macrocephaly (1/13; 7%) observed in infancy or enlarging fontanels (1/13; 7%). Suggestive anomalies in brain imaging contributed to the diagnosis in 3 children (3/13; 23%), one patient (4.5y) had delayed motor development, presented excessive fatigue during exercise and balance disorders.

In Group 3, diagnosed based on positive family history, the patient tested at the age of 9 complained about vertigo and vomiting (from the age of 7). Another one, at the time of diagnosis was asymptomatic, but further presented with slight balance disorders.

The frequency of encephalopathic crisis was 7%. It turns out that only one patient had such an incidence among 8 clinically diagnosed patients (from Group 2) and none among the patients diagnosed by newborn screening and selective testing carried out based on familiar risk. Further development of this patient (case 10) was normal, no balance disorders or seizures were observed during follow-up.

In this group, apart from head anomalies mentioned above, the most common during further observation were neurodevelopmental disorders, including: speech delay/deficits or poor language ability (4/13, 30%) and learning difficulties (2/13, 15%). Two patients were diagnosed with mild intellectual disability (2/13, 15%), 2 others suffered from seizures or epilepsy (2/13, 15%). Among 8 cases, 2 had no health problems (cases 10 mentioned above and 8). The latter however complains about periodic headaches.

### GC-MS data

Urinary concentrations of glutaric acid and 3-hydroxyglutaric acid were increased allowing, in accordance with clinical data, for glutaric aciduria type 1 recognition. The mean amount of glutaric acid was 1213.3 mmol/mol creatinine (18–4801 mmol/mol creatinine) and for 3-hydroxyglutaric acid – 52.6 mmol/mol creatinine (6.5–178 mmol/mol creatinine) (Suppl Table).

Among data available for 13 patients, 5 were low-excretors, defined by a urinary glutaric acid below 1000 mmol/mol creatinine (patients 1, 2, 5, 6, 10). The remaining 7 patients were high-excretors (Table 1) with glutaric acid excretions well above this value.

### MRI findings

Generally, in our Polish group the typical magnetic resonance findings of GA1 were the same as described in the previous publications (Kölker et al. 2003; Mohammad et al. 2015). These include: fluid collections over the convexities in the frontoparietal and frontotemporal areas, wide open sylvian fissures with probably hypoplastic operculum, bilateral lesions in the central gray matter (including the putamen, globus pallidus, head of the caudate nucleus, dentate nucleus and substantia nigra), and extensive the white matter abnormalities. The arcuate fibers and corpus callosum are usually spared although in 3 patients (6, 7 and 13) the corpus callosum discloses abnormal signal and has increased volume. Moreover, we also noticed that signal of normal myelinated optic radiation was preserved in all patients Fig. 3.

### *GCHD* mutational spectrum

As noted in Table 1, in our 13 cases, deriving from 11 nonconsanguineous families, pathogenic variants were identified on 25 alleles of *GCHD*. We found 9 homozygotes, 3 compound heterozygotes, and 1 monoallelic heterozygote (patient 10 without mutation on second allele, but in this case only selected *GCHD* exons were analysed and, since symptoms and biochemical results were consistent with the GA1 diagnosis, parents were not interested in further testing). All molecular variants were missense mutations, which have been previously described in correlation with GA1 phenotype.

The most frequent mutations was substitution c.1204C > T (p.Arg402Trp) noted in 13/25 (52%) alleles (including 2 sisters). This variant affects highly conserved amino acid and is located within acyl-CoA dehydrogenase, C-terminal domain. Moreover, two other changes were recurrent: c.700C > T (p.Arg234Trp) detected in 4/25 (16%) alleles (including 2 sisters) and c.1063C > T (p.Arg355Cys) observed in 3/25 (12%) alleles. Both of the variants also concern highly conserved amino acid and are located within acyl-CoA dehydrogenase/oxidase, N-terminal and middle domain and acyl-CoA dehydrogenase/oxidase C-terminal domain, respectively. The frequency of these three mutations in different population database is very low, <0.0005 for p.Arg402Trp and p.Arg355Cys and 0 for p.Arg234Trp substitution (Table 2). The remaining mutations were sporadic, present only in a few children.

**Table 2:** Minor allele frequency (MAF) of mutations identified in our 13 GA1 patients in comparison with frequency data in general population

Mutation	No. of mutant alleles	MAF	GnomAD	ExAc	ESP	POL
p.Arg402Trp	13	0.52	0.000258967	0.0002196	0.00046	0
p. Arg234Trp	4	0.16	0	0	0	0
p.Arg355Cys	3	0.12	0.0000323687	0.00003253	0	0
p.Arg313Trp	2	0.08	0.00000816413	0.00001626	0	0
p. Arg355His	1	0.04		0.00016		
p. Arg227Pro	1	0.04	0.00032476	0.0001789	0.00023	0
p. Ser259Pro	1	0.04	0	0	0	0

GnomAD - The Genome Aggregation Database (http://gnomad.broadinstitute.org/*);*

*ExAc -* The Exome Aggregation Consortium (http://exac.broadinstitute.org/);

ESP – The Exome Sequencing Project (https://esp.gs.washington.edu/drupal/)

POL – The Polish 400 exomes database (in-house database)

## Discussion

### Clinical data and GC-MS profile

The manifestation of glutaric aciduria type 1 is known to be variable (Haworth et al. 1991; Zafeiriou et al. 2000; Boy et al. 2017). Most cases are diagnosed with acute type, based on neurological sequelae after encephalopathic crises (as dystonic-dyskinetic disorder with typical imaging features). Most probably, the high frequency of this specific GA1 type results from its characteristic course. However, in approximately 25% of affected children, episodes of decompensation and encephalopathy are mild or absent. This applies to our group - the phenotype observed in the Polish patients presented herein is definitely mild, with an episode of metabolic decompression noted in only one patient (10, the low-excretor). Notably, nowadays the diagnosis through NBS is constantly increasing and becoming more frequent, that it also a reason that acute form is less frequently observed.

**Fig. 1:**
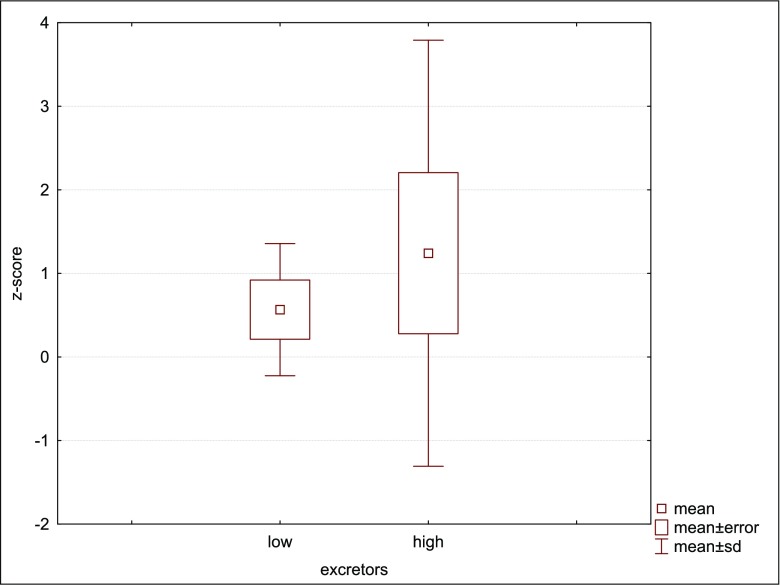
Mean head circumference in low-excretors and high-excretors

**Fig. 2:**
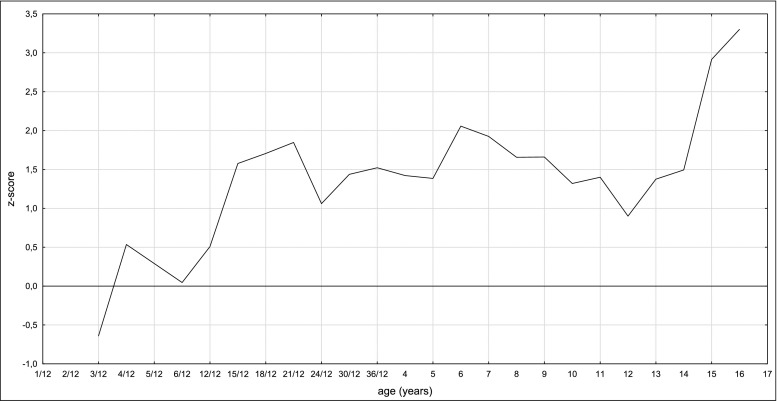
Mean z-score for head circumference

Of interest, contrary to other reports, where GA1 clinical outcome appears to be unrelated to biochemical or genotype data (Christensen et al. 2004; Zschocke et al. 2000), in our group some relation between heads circumference and urinary amount of glutaric acid may be noted (Fig. 1). In the high-excretors (Patients 3, 4, 7, 8, 9, 11, 12) head circumferences are in the upper normal range (5/7, 71%; the largest in 4 and 8) or their rapid increased can be noted (2/7, 28%). The latter was even an indicator for biochemical testing towards glutaric aciduria. Otherwise, the most rapid increases were noted in cases 2 and 12, sisters but with different glutaric acid excretion (Table 1). It does support the thesis that other factors than just the *GCDH* genotype determine the clinical course of GA1. Regarding the mean head circumferences, as one can note in Fig. 2 - an increase rate in the head circumferences with age, especially after the age of 10, was observed. Taking into account the positive predictive value of macrocephaly in glutaric aciduria, which is low, testing towards GA1 in every macrocephalic patient is not economically justified. Notwithstanding, following the proposed clinical methodology of selective screening for metabolic disorder, and based on our experience, we do recommend to check for GA1 in every case when rapid increase of the head circumference in infancy is observed (Lampret et al. 2015).

Another key issue concerning GA1 is whether the treatment (with low-protein diet that does not contain tryptophan and lysine, carnitine and riboflavin supplementation, has a significant impact on the clinical outcome. Based on our experience it seems rather not. The excretion of glutaric acid has indeed considerably reduced (see Table 2 in the supplementary data), but we have no data to prove that it correlates with the clinical outcome. We can only indicate that in 4 of our Polish patients who were diagnosed over 6 years (5, 6, 8, 13) neither metabolic incidence nor characteristic brain anomalies were reported. Two are asymptomatic and do not report any health problems. In 2 others mild intellectual disability was diagnosed and epilepsy in one, but since the etiology of these is definitely multifactorial, it does anyhow be related to glutaric aciduria as the cause. Moreover, as our group represents mild phenotype of GA1 with relatively late clinical onset (only 3 patients were diagnosed as newborns – one through NBS and two through positive family history), it is really difficult to assess the impact of the treatment on the clinical outcome. In other groups, for example those described by Couce et al., patients were characterized by severe course of the disease and underlying genotypes differs, hence these authors were able to conclude about the effects of dietary treatment (Couce et al. 2013).

### Neuroimaging (MRI findings)

Neuroimaging studies in glutaric aciduria type 1 typically manifest extracerebral accumulation of cerebrospinal fluid, atrophy in the frontotemporal regions and diffuse abnormalities in the white matter (Mohammad et al. 2015). Moreover, 20 to 30% of the affected have subdural haemorrhages or effusions (Haworth et al. 1991; Hoffmann et al. 1996). These however were not observed within our cohort. Apart from features mentioned below, MRI findings in Polish affected patients are generally in line with previous reports.

**Fig. 3:**
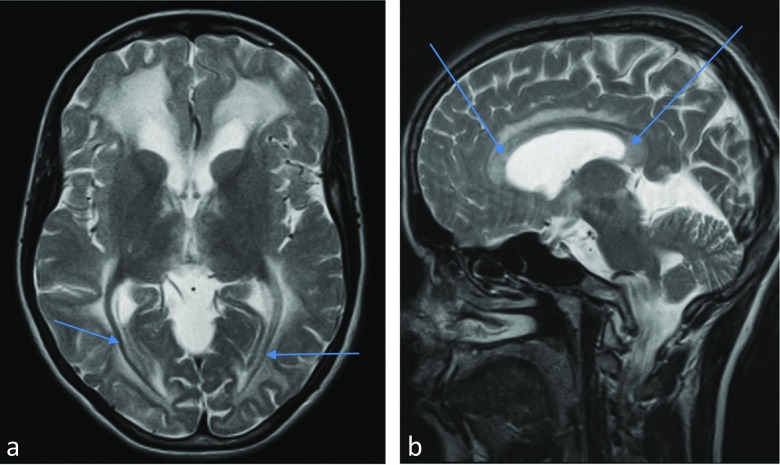
MRI - **a** Bilaterally preserved myelination of the optic radiation; **b** Abnormal signal of the corpus callosum

It is interesting, however, that in all of our patients the signal of normal myelinated optic radiation was preserved, regardless of the age of patients as well as diffuses signal abnormality in the white matter of both hemispheres. In 3 patients (cases 6, 7 and 13) the corpus callosum disclosed abnormal signal and had increased volume (Fig. 3). Two of them were homozygotes, and one was heterozygote for p.Arg402Trp. Moreover, in one of our patient - high-excretor, recognized based on selective GC-MS testing - congenital brain anomaly was noted (Table 1, case 9).

### Mutational data

More than two hundred molecular variants have been identified so far in *GCDH* gene the majority of which are missense mutations (www.hgmd.cf.ac.uk). According to Human Gene Mutation Database Professional 2018.3 (HGMD), 241 *GCDH* variants are currently mentioned (access 2018-10-15), including 225 disease-causing mutations and 16 of unknown clinical significance.

Most of mutations are private and specific for given families (Schwartz et al. 1998; Zschocke et al. 2000; Schmiesing et al. 2017). Certain mutations show predominance in specific populations e.g. the p.Ala421Val in the Amish community, the p.? (c.91 + 5G > T) mutation in Canadian Oji-Cree Indians, the p.Glu365Lys mutation in Irish Travelers, the p.Glu414Lys mutation in children of Lumbee heritage from North Carolina and the p.Ala293Thr mutation in black South Africans (Georgiou et al. 2014). The most common Caucasian mutation is the p.Arg402Trp, which accounts for 10–20% of alleles. It was expressed in *Escherichia coli* and resulted in the reduction of GCDH activity by >95%. One of the highest frequencies of the p.Arg402Trp mutation was found in patients of German (40%) and Spain origin (22%–28%) (Biery et al. 1996).

In our group, 7 different pathogenic variants were identified. All were previously reported. The mutational spectrum of *GCDH* gene observed in the present study indicates genetic homogeneity among patients of Polish ancestry. The p.Arg402Trp variant was observed in above 50% alleles in our group, which is significantly more common compared to the other reported GA1 relatives and to the general population. The most frequent is c.1204C > T (p.Arg402Trp) noted in 13 alleles (in 8 patients), followed by c.1063C > T (p.Arg355Cys) and c.700C > T (p.Arg234Trp) in 3 and 2 alleles respectively.

Despite the fact that most our patients carry p.Arg402Trp variant, being the most frequent in European population, the frequency in our cohort is much higher (10–20% vs. 53%). Moreover, related phenotypes observed in Polish patients are definitely mild, regardless of the amount of the glutaric and 3-hydroxyglutaric acids urinary excretion. This may support the existence of the other genetic or (and) non-genetic factors contributing to clinical outcome, as well as suggest at least partial specificity of our population. These however should be verified in the future studies.

Regarding genotype-phenotype, we did not observe unambiguous correlation. Nevertheless, we would like to draw attention to certain features noted in our homozygotes p.Arg402Trp (five patients – 3, 4, 7, 11, 13) and p.Arg234Trp (two patients – 2 and 12). In the p.Arg402Trp group: only one was the low-excretor, and 3 of 5 cases – contrary to our other patients - had learning difficulties and(or) intellectual disability. It is also worth noting that in 3 cases within this group specific (mentioned above) changes in corpus callosum and white matter were observed. Whereas in both sisters with homozygous p.Arg234Trp variants a balance disorder and unsteady gait were present (as in only ones in our group) only one was the high-excretor. Of course, we are aware of the small size of these two groups, but we hope that this data will be tested in further research.

## Conclusions

Based on the presented results we conclude that clinical course of glutaric aciduria type 1 in Polish cohort is mild what might suggest some specificity of our population, especially due to the high predominance of the p.Arg402Trp variant in *GCDH* gene. Despite the normal development observed in most of our patients, we proposed to refer all patients for appropriate therapies, especially regarding speech deficits and decreased fine motor skills. Larger head circumference does correlate to some extent with gluratic and 3-hydroxyglutaric acids urinary excretion. Although macrocephaly has low positive predictive value for GA1, we would suggest keeping it in mind in the context of GA. Finally, the p.Arg402Trp variant may predispose to larger head circumference and specific brain anomalies, while p.Arg234Trp variant may result in a balance disorder.

## Electronic supplementary material

ESM 1 (DOCX 15 kb)
