# Supplementary material for: Mild phenotype of glutaric aciduria type 1 in polish patients – novel data from a group of 13 cases
Source: Metab Brain Dis. 2018 Dec 20;34(2):641–9. doi: 10.1007/s11011-018-0357-5 (PMC6428789; doi:10.1007/s11011-018-0357-5)
Supplement: Supplementary file 1 — (DOCX 15 kb) [file 11011_2018_357_MOESM1_ESM.docx]

Supp. Table. GC-MS results of 13 Polish patients with glutaric aciduria type I (high-excretors are marked in bold)

| Patient | Glutaric acid  (mmol/mol creatinine)  n<10 | 3-hydroksyglutaric acid  (mmol/mol creatinine)  n<5 | Acylocarnitines  C5DC  Norm given in the bracket  Na- not assesed |
| --- | --- | --- | --- |
| 1m | 22 | 6,6 | na |
|  | 18 | 11 |  |
|  | 21 | 10 |  |
| 2/f  (sister of 12) | 800 | 18 | 1,8 umol/L  n =0,3 |
|  | 2524 | 30 |  |
|  | 2991 | 57 |  |
|  | 3801 | 73 |  |
| **3/f**  (sister of 13) | 2636 | 48 | 0,63nmol/mL  n=0,13 |
|  | 1544 | 77 |  |
|  | 603 | 48 |  |
| **4/m** | 4801 | 14 | 6,27 µM  n=0,23 |
|  | 1832 | 28 |  |
|  | 442 | 75 |  |
|  | 240 | 44 |  |
|  | 409 | 53 |  |
|  | 294 | ? |  |
|  | 441 | 31 |  |
|  | 632 | 38 |  |
| 5/m | 773 | 59 | 0,22 nmol/mL  n=0,13 |
|  | 857 | 41 |  |
| 6/f | 546 | 60 | 1,50 µM  n=0,23 |
| **7/f** | 3766 | 50 | 0,52 nmol/mL  n=0,13 |
|  | 2606 | 47 |  |
|  | 1043 | 53 |  |
| **8/f** | 1719 | 92 | 0,21 umol/mL  n =0,13 |
|  | 632 | 31 |  |
|  | 846 | 6,5 |  |
|  | 685 | 51 |  |
| **9/f** | 1741 | 60 | 5,92 µM  n=0,23 |
|  | 559 | 69 |  |
|  | 681 | 44 |  |
|  | 913 | 55 |  |
|  | 348 | 38 |  |
| 10/f | 2703 | 77 | 3,4 µM  n=0,23 |
|  | 1514 | 178 |  |
|  | 1287 | 51 |  |
|  | 979 | 32 |  |
|  | 385 | 67 |  |
|  | 629 | 121 |  |
| **11/m** | 1374 | 79 | 0,53 umol/L  n =0,3 |
|  | 956 | 88 |  |
| **12/f** | 2343 | 36 | na |
|  | 641 | 37 |  |
| 13/f  (sister of 3) | 806 | 58 | 3,4 umol/mL  n =0,13 |
|  | 583 | 61 |  |
|  | 571 | 67 |  |
|  | 487 | 42 |  |
